# Beyond the Tumour: An Endocrine Pitfall of Immunotherapy

**DOI:** 10.7759/cureus.91766

**Published:** 2025-09-07

**Authors:** Cho May Than, Kyaw Maung Maung, Ye Win, Nyi Htwe

**Affiliations:** 1 Acute Medicine, United Lincolnshire Teaching Hospital Trust, Boston, GBR; 2 Internal Medicine, University of Medicine (1) Yangon, Yangon, MMR; 3 Endocrinology and Diabetes, United Lincolnshire Teaching Hospital Trust, Boston, GBR

**Keywords:** adrenal insufficiency (ai), advanced melanoma, hypophysitis immune checkpoint inhibitors (icis), immune checkpoint inhibitors, pembrolizumab side effect

## Abstract

Immune checkpoint inhibitors (ICIs) such as pembrolizumab have transformed the treatment of advanced melanoma but are associated with immune-related adverse events (irAEs), including endocrine dysfunction such as adrenal insufficiency, which may be permanent and life-threatening. We report the case of a 62-year-old woman with Stage III BRAF-negative cutaneous melanoma of the left upper arm who developed pembrolizumab-induced adrenal insufficiency after three cycles of adjuvant therapy. She presented with gastrointestinal symptoms and systemic inflammation, and subsequent evaluation revealed cortisol <25 nmol/L with undetectable adrenocorticotropic hormone, consistent with secondary adrenal insufficiency. Pembrolizumab was discontinued, and she remains clinically stable on hydrocortisone replacement with surveillance imaging showing no evidence of recurrence. This case underscores the importance of vigilance for irAEs in patients receiving ICIs, as adrenal insufficiency can present with nonspecific symptoms and may be misdiagnosed without timely biochemical evaluation. Regular monitoring and early endocrine involvement are essential for accurate diagnosis and effective long-term management.

## Introduction

Immune checkpoint inhibitors (ICIs) such as pembrolizumab have revolutionised the management of malignant melanoma. Pembrolizumab blocks the programmed death-1 (PD-1) receptor, enhancing antitumour immunity by inhibiting T-cell inactivation [1]. However, ICIs can trigger immune-related adverse events (irAEs), including endocrine dysfunctions like hypophysitis, thyroiditis, and adrenalitis [2]. The incidence of hypophysitis is more common with CTLA-4 inhibitors, but anti-PD-1 agents like pembrolizumab can also cause this rare adverse effect [3]. Hypophysitis with isolated adrenocorticotropic hormone (ACTH) deficiency, though uncommon, poses a diagnostic challenge due to its non-specific presentation [4].

Recognising and managing such complications early is crucial to prevent morbidity and mortality. Clinical suspicion should be heightened in patients on ICIs presenting with symptoms like fatigue, anorexia, hypotension, or gastrointestinal disturbances, or skin changes, including hyperpigmentation or darkening of the skin [5-7].

## Case presentation

A 62-year-old woman with a background of type 2 diabetes and no known drug allergies was initially diagnosed with Stage IIa cutaneous superficial spreading malignant melanoma of the left forearm (Breslow thickness 3.1 mm, 3/mm²). She declined sentinel lymph node biopsy at the time and remained under surveillance for several years. She later presented with recurrent in-transit metastases in the left upper arm, confirmed on biopsy.

She underwent wide local excision, which revealed two in-transit metastases with close margins. A further excision confirmed no residual disease. Staging CT and PET/CT scans showed no evidence of distant metastasis. Based on these findings, she was reclassified as Stage III and commenced on adjuvant pembrolizumab, administered every six weeks with a planned duration of one year.

After three cycles of immunotherapy, she developed low-grade diarrhoea, followed by an acute admission with fever, loose stools, fatigue, and raised inflammatory markers (CRP 121 mg/L; WCC 417 ×10⁹/L). A working diagnosis of infective colitis was made, and she was treated empirically. However, due to her recent exposure to immune checkpoint inhibitors, immune-related colitis remained a possible differential. She was commenced on a short course of prednisolone.

During this admission, a low early morning cortisol (16 nmol/L) and undetectable ACTH (<5 pmol/L), as shown in Table 1, led to the diagnosis of secondary adrenal insufficiency, likely immune-related hypophysitis. Pituitary profile, including thyroid-stimulating hormone (TSH), luteinizing hormone (LH), follicle-stimulating hormone (FSH), insulin-like growth factor 1 (IGF-1), estradiol, and prolactin, was otherwise normal.

**Table 1:** Serial laboratory investigations with corresponding normal reference ranges TSH, thyroid-stimulating hormone; LH, luteinizing hormone; FSH, follicle-stimulating hormone; IGF-1, insulin-like growth factor 1; ACTH, adrenocorticotropic hormone; HbA1c, glycated haemoglobin; GFR, glomerular filtration rate; AST, aspartate aminotransferase; ALT, alanine aminotransferase; γGT, gamma-glutamyl transferase; LDH, lactate dehydrogenase; CRP, C-reactive protein; MCH, mean corpuscular haemoglobin; MCHC, mean corpuscular haemoglobin concentration; NA, not applicable; NC, not calculated

Test name	9/29/2023	9/17/2024	9/26/2024	11/8/2024	12/20/2024	1/31/2025	2/10/2025	3/15/2025	3/16/2025	3/17/2025	3/18/2025	3/19/2025	7/9/2025	Normal range
Fasting glucose	–	–	7.1 mmol/L	6.5 mmol/L	8.3 mmol/L	7.7 mmol/L	–	7.2 mmol/L	–	–	–	–	–	3.0-6.0 mmol/L
HbA1C (IFCC)	54 mmol/mol	–	–	–	52 mmol/mol	54 mmol/mol	–	–	–	–	–	–	56 mmol/mol	20-41 mmol/mol
FSH	NA	NA	NA	NA	NA	NA	45.2 IU/L	NA	NA	NA	NA	NA	NA	30 – 118 IU/L (for Post-menopausal)
LH	NA	NA	NA	NA	NA	NA	16 IU/L	NA	NA	NA	NA	NA	NA	16 – 66 IU/L (for Post-menopausal)
17 beta oestradiol	NA	NA	NA	NA	NA	NA	<18 pmol/L	NA	NA	NA	NA	NA	NA	Less than 118 pmol/L (for Post-menopausal)
Prolactin	NA	NA	NA	NA	NA	NA	414 mIU/L	NA	NA	NA	NA	NA	NA	102-496 mIU/L
Early morning cortisol	–	–	–	–	17 nmol/L	26 nmol/L	16 nmol/L	–	–	–	–	–	–	100-350 nmol/L
Short Synacthen test, 30-minute cortisol level	NA	NA	NA	NA	NA	NA	192 nmol/L	NA	NA	NA	NA	NA	NA	≥ 430 nmol/L indicates normal adrenal gland response
ACTH	NA	NA	NA	NA	NA	NA	<5 ng/L	NA	NA	NA	NA	NA	NA	7.2-63.3 ng/L
Growth factor IGF-1	NA	NA	NA	NA	NA	NA	NA	NA	NA	NA	NA	NA	NA	7.8-23.5 nmol/L
TSH	1.3 mU/L	–	–	–	1.2 mU/L	1.3 mU/L	2.1 mU/L	1.9 mU/L	–	–	–	–	2 mU/L	0.27-4.5 mU/L
Free T4	–	–	–	–	13.7 pmol/L	10.2 pmol/L	11.2 pmol/L	12.2 pmol/L	–	–	–	–	–	11.0-23.0 pmol/L
Free T3	–	–	–	–	4.2 pmol/L	–	–	–	–	–	–	–	–	3.5 – 6.5 pmol/L
Sodium	140 mmol/L	140 mmol/L	139 mmol/L	139 mmol/L	139 mmol/L	142 mmol/L	–	134 mmol/L	133 mmol/L	139 mmol/L	138 mmol/L	138 mmol/L	141 mmol/L	133-146 mmol/L
Potassium	5.5 mmol/L	4.7 mmol/L	4.5 mmol/L	4.5 mmol/L	5.1 mmol/L	5.2 mmol/L	–	4.4 mmol/L	3.7 mmol/L	4.4 mmol/L	4.4 mmol/L	3.8 mmol/L	4.4 mmol/L	3.5-5.3 mmol/L
Urea	3.9 mmol/L	4.1 mmol/L	3.9 mmol/L	4 mmol/L	5.7 mmol/L	4.4 mmol/L	–	5.1 mmol/L	5.7 mmol/L	5.2 mmol/L	5.6 mmol/L	5 mmol/L	5.8 mmol/L	2.5-7.8 mmol/L
Creatinine	69 umol/L	64 umol/L	65 umol/L	74 umol/L	73 umol/L	67 umol/L	–	86 umol/L	79 umol/L	53 umol/L	78 umol/L	77 umol/L	72 umol/L	45-84 umol/L
GFR (EPI-2009)	82 ml/min	89 ml/min	87 ml/min	75 ml/min	76 ml/min	84 ml/min	NC	62 ml/min	69 ml/min	>90 ml/min	70 ml/min	71 ml/min	77 ml/min	90-200 ml/min
Magnesium	–	0.86 mmol/L	0.89 mmol/L	0.92 mmol/L	0.85 mmol/L	0.9 mmol/L	–	–	–	–	–	0.91 mmol/L	–	0.70-1.00 mmol/L
Bilirubin	10 umol/L	7 umol/L	8 umol/L	8 umol/L	6 umol/L	4 umol/L	–	7 umol/L	7 umol/L	<3 umol/L	6 umol/L	7 umol/L	5 umol/L	0-21 umol/L
AST	–	–	20 U/L	20 U/L	17 U/L	18 U/L	–	–	–	11 U/L	–	–	–	0-32 U/L
ALT	25 U/L	23 U/L	25 U/L	21 U/L	17 U/L	18 U/L	–	14 U/L	15 U/L	12 U/L	11 U/L	14 U/L	12 U/L	0-33 U/L
Alkaline phosphatase	56 U/L	61 U/L	60 U/L	63 U/L	63 U/L	57 U/L	–	55 U/L	52 U/L	46 U/L	43 U/L	47 U/L	65 U/L	30-130 U/L
Gamma GT	–	–	–	–	–	–	–	–	–	10 U/L	–	–	–	5.9-42 U/L
Total protein	70 g/L	72 g/L	75 g/L	74 g/L	73 g/L	67 g/L	–	73 g/L	63 g/L	57 g/L	58 g/L	66 g/L	70 g/L	60-80 g/L
Albumin	37 g/L	39 g/L	38 g/L	38 g/L	37 g/L	33 g/L	–	34 g/L	32 g/L	28 g/L	28 g/L	33 g/L	35 g/L	35-50 g/L
Globulin	33 g/L	33 g/L	37 g/L	36 g/L	36 g/L	34 g/L	–	–	31 g/L	29 g/L	30 g/L	33 g/L	35 g/L	20-40 g/L
Calcium	–	–	2.45 mmol/L	2.5 mmol/L	2.52 mmol/L	2.37 mmol/L	–	2.49 mmol/L	2.17 mmol/L	2.23 mmol/L	2.29 mmol/L	2.35 mmol/L	–	2.2-2.6 mmol/L
Adjusted calcium	–	–	2.48 mmol/L	2.53 mmol/L	2.56 mmol/L	2.47 mmol/L	–	2.57 mmol/L	2.28 mmol/L	2.39 mmol/L	2.45 mmol/L	2.45 mmol/L	–	2.20-2.60 mmol/L
Phosphate	–	–	1.26 mmol/L	1.53 mmol/L	1.33 mmol/L	1.49 mmol/L	–	–	1.06 mmol/L	1.13 mmol/L	0.99 mmol/L	1.15 mmol/L	–	0.80-1.50 mmol/L
LDH	–	–	139 U/L	133 U/L	142 U/L	148 U/L	–	–	123 U/L	–	–	–	–	135-214 U/L
CRP	–	–	–	–	–	–	–	8 mg/L	66 mg/L	121 mg/L	44 mg/L	25 mg/L	–	0-5 mg/L
Haemoglobin	130 g/L	135 g/L	132 g/L	–	126 g/L	130 g/L	–	132 g/L	123 g/L	116 g/L	115 g/L	121 g/L	124 g/L	117-149 g/L
White cell count	9.4 x 10*9/L	7.1 x 10*9/L	7.5 x 10*9/L	–	9.7 x 10*9/L	7.3 x 10*9/L	–	7.2 x 10*9/L	8.6 x 10*9/L	6.1 x 10*9/L	9 x 10*9/L	9.2 x 10*9/L	8.2 x 10*9/L	4.3-11.2 x 10*9/L
Platelets	398 x 10*9/L	424 x 10*9/L	400 x 10*9/L	–	416 x 10*9/L	350 x 10*9/L	–	428 x 10*9/L	363 x 10*9/L	364 x 10*9/L	399 x 10*9/L	432 x 10*9/L	417 x 10*9/L	150-400 x 10*9/L
Red cell count	4.4 x 10*12/L	4.6 x 10*12/L	4.49 x 10*12/L	–	4.34 x 10*12/L	4.45 x 10*12/L	–	4.57 x 10*12/L	4.28 x 10*12/L	3.96 x 10*12/L	3.97 x 10*12/L	4.22 x 10*12/L	4.26 x 10*12/L	3.85-5.15 x 10*12/L
Mean cell volume	88 fL	87 fL	87 fL	–	87 fL	90 fL	–	86 fL	88 fL	85 fL	89 fL	89 fL	90 fL	81-97 fL
Haematocrit	0.387	0.401	0.391	–	0.379	0.401	–	0.393	0.376	0.337	0.354 pg	0.375	0.385	0.347-0.445
MCH	29.5 pg	29.3 pg	29.4 pg	–	29 pg	29.2 pg	–	28.9 pg	28.7 pg	29.3 pg	29 pg	28.7 pg	29.1 pg	26.9-33 pg
MCHC	336 g/L	337 g/L	338 g/L	–	332 g/L	324 g/L	–	336 g/L	327 g/L	344 g/L	325 g/L	323 g/L	322 g/L	320-359 g/L
Neutrophils	7.02 x 10*9/L	3.91 x 10*9/L	4.54 x 10*9/L	–	5.6 x 10*9/L	3.44 x 10*9/L	–	5.4 x 10*9/L	5.46 x 10*9/L	4.37 x 10*9/L	4.76 x 10*9/L	3.78 x 10*9/L	3.02 x 10*9/L	2.1-7.4 x 10*9/L
Lymphocytes	1.55 x 10*9/L	2.38 x 10*9/L	2.03 x 10*9/L	–	3.13 x 10*9/L	2.74 x 10*9/L	–	1.3 x 10*9/L	2.27 x 10*9/L	1.19 x 10*9/L	3.51 x 10*9/L	4.51 x 10*9/L	4.15 x 10*9/L	1.0-3.6 x 10*9/L
Monocytes	0.61 x 10*9/L	0.52 x 10*9/L	0.52 x 10*9/L	–	0.64 x 10*9/L	0.83 x 10*9/L	–	0.38 x 10*9/L	0.73 x 10*9/L	0.55 x 10*9/L	0.69 x 10*9/L	0.78 x 10*9/L	0.72 x 10*9/L	0.3-1.0 x 10*9/L
Eosinophils	0.21 x 10*9/L	0.26 x 10*9/L	0.3 x 10*9/L	–	0.22 x 10*9/L	0.25 x 10*9/L	–	0.07 x 10*9/L	0.04 x 10*9/L	0 x 10*9/L	0.02 x 10*9/L	0.03 x 10*9/L	0.22 x 10*9/L	0.02-0.5 x 10*9/L
Basophils	0.05 x 10*9/L	0.07 x 10*9/L	0.06 x 10*9/L	–	0.06 x 10*9/L	0.07 x 10*9/L	–	0.03 x 10*9/L	0.07 x 10*9/L	0.01 x 10*9/L	0.03 x 10*9/L	0.05 x 10*9/L	0.07 x 10*9/L	0.02-0.1 x 10*9/L

**Figure 1:**
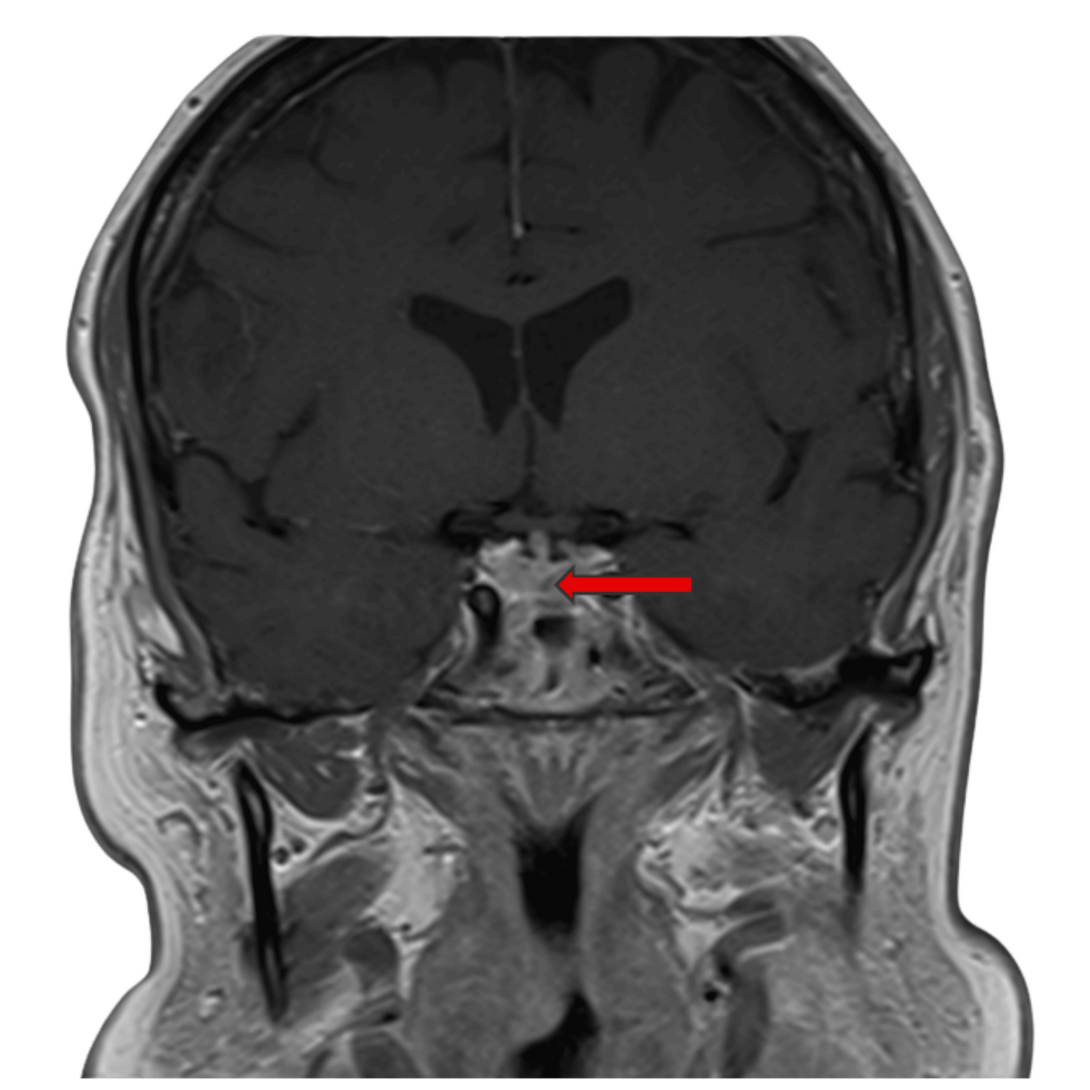
Coronal post-contrast pituitary MRI showing a 2 mm hypoenhancing microadenoma (MA) in the anterior lobe

**Figure 2:**
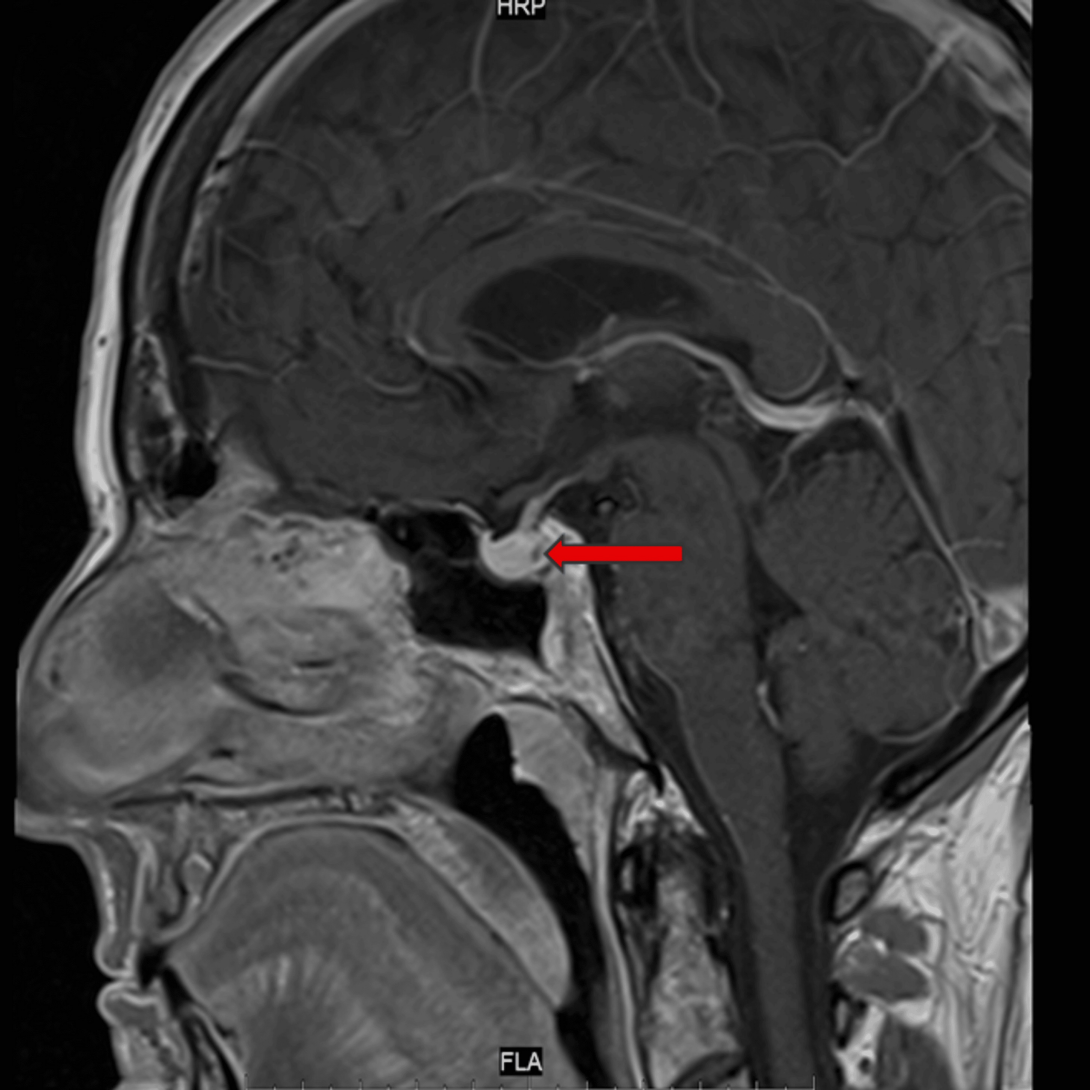
Sagittal post-contrast pituitary MRI showing a 2 mm hypoenhancing microadenoma (MA) in the anterior lobe

MRI pituitary imaging demonstrated a 2 mm anterior pituitary lesion, radiologically consistent with a microadenoma, without any involvement of the pituitary stalk, optic chiasm, or surrounding structures (Figures 1, 2). Pembrolizumab was permanently discontinued, and she was transitioned to physiological hydrocortisone replacement.

At subsequent endocrine clinic review, she was clinically stable, compliant with her steroid regimen (10 mg morning, 5 mg midday, 5 mg evening), and well-educated on sick day rules. She carried a medic-alert card and an emergency injection kit. Plans were made to consider tapering her steroid dose after several months of stability, pending clinical reassessment. She remains under oncology surveillance with no radiological evidence of melanoma recurrence.

Differential diagnosis

The patient’s acute presentation with gastrointestinal symptoms, systemic inflammation, and fever raised several potential diagnoses. Infective colitis was supported by elevated C-reactive protein (CRP) and white cell count (WCC), and a favourable response to empirical treatment. However, immune-mediated ileocolitis could not be excluded, given her recent pembrolizumab exposure. The absence of worsening symptoms or need for prolonged immunosuppression made this less likely. Sepsis of unknown origin was initially suspected but became less probable following negative cultures and clinical improvement.

Ultimately, the hallmark findings of low serum cortisol and suppressed ACTH pointed to secondary adrenal insufficiency due to immune checkpoint inhibitor-induced hypophysitis. Normal thyroid function and other pituitary hormones excluded other forms of panhypopituitarism, and the MRI findings of a stable 2 mm microadenoma were incidental and not felt to contribute to the hormonal axis disturbance.

Treatment

Upon diagnosis of secondary adrenal insufficiency, the patient was transitioned from prednisolone to hydrocortisone replacement therapy at a regimen of 10 mg in the morning, 5 mg at midday, and 5 mg in the evening. She was comprehensively educated on adrenal crisis prevention, steroid sick day rules, and emergency injection use. She received a medic-alert card and a hydrocortisone emergency kit. Pembrolizumab was permanently discontinued due to the immune-related endocrinopathy. Supportive care during her acute admission included intravenous fluids, infection screening, and symptom monitoring.

Follow-up

At her endocrine clinic review, she reported clinical stability, adherence to therapy, and understanding of self-management protocols. As she remained asymptomatic and without evidence of recurrence, a plan was made to trial a dose reduction of hydrocortisone after several months, with reassessment of adrenal axis function at a later stage. Adrenal function is typically re-evaluated every three to six months during follow-up, with adjustments guided by clinical status and biochemical results. A telephone follow-up was scheduled to monitor long-term endocrine function. She continues under oncology follow-up with no evidence of melanoma recurrence on recent surveillance imaging.

## Discussion

Pembrolizumab-associated endocrine irAEs have been increasingly recognised with widespread ICI use [8]. Secondary adrenal insufficiency due to isolated ACTH deficiency is rare and challenging to diagnose due to non-specific symptoms [5,6]. Fatigue, anorexia, diarrhoea, and hypotension should prompt evaluation for adrenal dysfunction, especially in patients receiving ICIs [9,10].

Our patient presented with a sepsis-like picture and GI symptoms, which may overlap with immune-mediated colitis or infection. However, biochemical evidence of adrenal insufficiency and suppressed ACTH supported ICI-induced hypophysitis with isolated ACTH deficiency. Literature reports similar presentations across various malignancies treated with pembrolizumab, including lung and breast cancer [11-13].

Long-term corticosteroid replacement is often required, and adrenal recovery may be delayed or absent [3,14]. Education on sick day rules and emergency steroid administration is critical to patient safety [15,16]. Some patients may remain steroid-dependent for life, particularly if irreversible pituitary damage has occurred [17,18].

Recent pharmacovigilance reports suggest under-recognition of pembrolizumab-induced hypophysitis, with delayed diagnosis contributing to morbidity [19]. Regular cortisol monitoring and patient education during ICI therapy may help mitigate complications.

## Conclusions

Pembrolizumab-induced adrenal insufficiency is a rare but serious complication that may mimic infection or colitis. High clinical suspicion, timely hormonal evaluation, and appropriate management are essential. This case highlights the need for education, regular follow-up, and a multidisciplinary approach to optimise outcomes.
